# Endometriosis phenotypes and staging in relation to lipid biomarkers: Findings from the ENDO Cohort Study

**DOI:** 10.1016/j.jogoh.2025.103087

**Published:** 2025-12-15

**Authors:** Karen C. Schliep, May Shaaban, Emmanuel Adediran, Anna Z. Pollack, Kathryn M. Rexrode, Rachael Hemmert, Madeline Paulsen, Jessica Treidl, Hediyeh Baradaran, Jennifer J. Majersik, Michael W. Varner, C. Matthew Peterson, Joseph B. Stanford, Jenna R. Krall, Jessica M. Page, Leslie V. Farland

**Affiliations:** a Department of Family and Preventive Medicine, University of Utah, Salt Lake City, UT, USA; b Department of Global and Community Health, College of Public Health, George Mason University, Fairfax, VA, USA; c Division of Women’s Health, Department of Medicine, Brigham and Women’s Hospital, Boston MA, USA; d Department of Radiology, Salt Lake City, UT, USA; e Department of Neurology, University of Utah, Salt Lake City, UT, USA; f Department of Obstetrics and Gynecology, University of Utah, Salt Lake City, UT, USA; g Division of Maternal-Fetal Medicine, Intermountain Health Care, Murray, UT, USA; h Department of Epidemiology and Biostatistics and Department of Obstetrics and Gynecology, University of Arizona, Tucson, AZ, USA

**Keywords:** Hyperlipidemia, Dyslipidemia, Cardiovascular disease, Endometriosis staging, Endometriosis typology

## Abstract

**Background:**

Endometriosis has been linked to cardiometabolic alterations, but whether these associations vary by disease severity or phenotype is unclear. We examined lipid profiles across endometriosis diagnosis, stage, and typology.

**Material and methods:**

Data came from 476 women in the NICHD ENDO cohort. Endometriosis was confirmed laparoscopically and staged using the rASRM criteria (*I*–IV). Typology was categorized as superficial endometriosis (SE), ovarian endometrioma (OE), deep infiltrating endometriosis (DE), and OE+DE. We compared endometriosis status, stage (I/II vs III/IV), and typology to no endometriosis using adverse lipid thresholds (total cholesterol ≥200 mg/dL, HDL <50 mg/dL, LDL ≥100 mg/dL, triglycerides ≥175 mg/dL, non-HDL ≥130 mg/dL, VLDL ≥30 mg/dL, ApoA1 <125 mg/dL, and ApoB ≥120 mg/dL). Adjusted prevalence ratios (aPR) and 95 % CIs were estimated via generalized linear models, controlling for age, race/ethnicity, BMI, income, marital status, and serum cotinine.

**Results:**

Endometriosis diagnosis alone was not associated with adverse lipid profiles. In contrast, moderate/severe disease showed higher prevalence of elevated triglycerides (aPR= 2.27; 95 % CI: 1.18,4.35) and VLDL (aPR= 2.41; 95 % CI: 1.50, 3.85). Typology revealed stronger patterns: OE and OE+DE were associated with adverse profiles across multiple markers (aPRs 1.59–4.09), particularly ApoB and triglycerides. Minimal/mild disease and SE were not associated.

**Conclusions:**

The metabolic signal was phenotype-driven rather than diagnosis-driven, with severe stage and OE/OE+DE showing clear associations with adverse lipid profiles. These findings suggest lipid profiles may serve as markers of phenotype severity or shared biological milieu. Replication in larger cohorts is needed.

## 1. Introduction

Endometriosis is a benign, chronic gynecological condition characterized by the presence of endometrial-like tissue outside the uterine cavity. It is estimated that endometriosis affects approximately 11 % of women of reproductive age and is most commonly associated with dysmenorrhea, pelvic pain, and infertility [1]. There is emerging literature suggesting that women with endometriosis may be at elevated risk for other chronic conditions later in life, including cardiovascular disease (CVD). Although the mechanisms underlying these associations are not fully understood, it has been hypothesized that aberrant systemic changes may be an overlapping cause or consequence of endometriosis and risk of CVD.

CVD is the leading cause of death in US women [2]. Previous research has suggested that women with endometriosis may have an increased risk of developing CVD, such as heart attack and stroke, later in life [3]. Women with endometriosis may have a higher risk of CVD precursors, including hypertension [4], adverse lipid profile [4-7], arterial stiffness [8], and subclinical atherosclerosis [9], leading to CVD. Emerging research suggests a bidirectional association between endometriosis and dyslipidemia [4], with several mechanistic pathways potentially at work including systemic inflammation with endothelial dysfunction, hormonal dysregulation, and shared genetic predispositions [4,10].

This adverse CVD profile may be detectable at the time of endometriosis diagnosis. However, few studies have investigated lipid profiles for women with endometriosis at the time of diagnosis and very few studies have investigated endometriosis lesion location and stage in relation to dyslipidemia biomarkers. We investigated the association between endometriosis and concentrations of total cholesterol, high-density lipoprotein (HDL), low-density lipoprotein (LDL), triglycerides, non-HDL, VLDL, Apolipoprotein A-1 (ApoA1), Apolipoprotein B (ApoB); ApoB/ApoA1 ratio; and lipoprotein a (Lp[a]). Endometriosis was classified by staging and typology.

## 2. Materials and methods

### 2.1. Study population

The study population included women who enrolled in the Endometriosis, Natural History, Diagnosis, and Outcomes (ENDO) Study from the Utah site who had stored biospecimens available for assay (*n* = 476) [1]. In brief, eligible women were currently menstruating, aged 18–44 years, and without a history of visually confirmed endometriosis. Additionally, they could have no injectable hormonal treatment within the past two years. The ENDO Study population comprised an operative and population cohort. The original purpose of the ENDO study was to 1) to estimate the scope and magnitude of endometriosis at both the clinical and population levels; and 2) to assess the relation of endocrine disrupting chemicals and risk of gynecologic pathology including endometriosis.

The operative cohort was scheduled to undergo a diagnostic and/or therapeutic laparoscopy or laparotomy irrespective of clinical indication at one of 5 participating hospital surgical centers located in Utah 2007–2009. Surgical indications for laparoscopy/laparotomy included pelvic pain (44 %), pelvic mass (16 %), irregular menses (13 %), fibroids (10 %), tubal ligation (10 %), and infertility (7 %).

The population cohort had the same inclusion/exclusion criteria as the operative cohort including currently menstruating, to ensure they were at risk for developing endometriosis. However, women in the population cohort were not scheduled to undergo a diagnostic and/or therapeutic laparoscopy or laparotomy. Rather, the population cohort came from the geographic catchment areas for the participating surgical centers of the operative cohort. The population cohort agreed to undergo a pelvic MRI for diagnosing endometriosis.

### 2.2. Data collection

Standardized data collection included a baseline personal interview to assess sociodemographic, lifestyle, and health history, anthropometric assessment; operative and MRI reports; and collection of biospecimens, including serum [1]. Women in both cohorts completed the interview prior to surgery or MRI.

### 2.3. Exposure

#### 2.3.1. Endometriosis, operative cohort

The exposure of interest was visualized endometriosis diagnosis during surgery (yes/no) along with endometriosis stage and typology. For endometriosis, all surgeons participating in the ENDO study were trained in the diagnosis and staging of endometriosis. The surgeons completed a standardized operative report immediately after surgery to capture the degree of endometriosis and gynecologic and pelvic pathology, including endometriosis, uterine fibroids, pelvic adhesions, benign ovarian cysts, neoplasms, and congenital Mullerian anomalies.

Endometriosis staging was categorized using the revised American Society for Reproductive Medicine (rASRM) disease stage. The rASRM uses a weighted point score to categorize endometriosis staging: stage I, minimal (scores 1–5); stage II, mild (scores 6–15); stage III, moderate (scores 16–40); and stage IV, severe (scores >40). Our prior research showed substantial agreement between gynecologic surgeons on endometriosis diagnosis and staging after viewing digital images and after additionally viewing operative reports [11].

Typology was assessed via the rASRM standardized form for women whose rASRM form had information on lesion location, depth, and size (*n* = 180 [95 %] out of the 190 women with an endometriosis diagnosis). Women with superficial lesions on the ovary or peritoneum were categorized as having SE. Deep lesions were categorized separately and later combined; deep lesions (>5 mm invasion), on the peritoneum or obliteration of posterior cul-de-sac, were considered to be DE; and deep lesions of any size noted in the ovary were considered to be OE. These two categories of deep lesions, (i.e., deep ovarian and deep peritoneal lesions) were looked at combined: OE + DE. Women without information on lesion location, size, and depth (*n* = 10) were assumed to have SE.

“No endometriosis” served as the comparison group for diagnosis, staging, and typology analyses. “No endometriosis” included women with no endometriosis but with other gynecological conditions and women with no endometriosis and a normal pelvis. A sensitivity analysis was conducted assessing these two reference groups separately.

#### 2.3.2. Endometriosis, population cohort

In the population cohort, participation required a willingness to undergo a pelvic MRI to identify endometriosis. All pelvic MRIs were performed using either a Siemens Avanto or Espree 1.5 Tesla scanner and a U.S. FDA approved protocol for pelvic imaging, and completed standardized data collection instruments [1]. All images were double read for research purposes, first by the initial and, subsequently, by a second radiologist.

#### 2.3.3. Outcome: lipid biomarkers

Blood samples were collected (2007–2009) prior to surgery (surgical cohort) or MRI (population cohort). Serum samples were aliquoted and frozen at −80-degree Celsius. Lipid markers were assayed using banked serum specimens by ARUP laboratories (Salt Lake City, Utah) in 2024. A quantitative enzymatic assay was used for the lipid panel (total; HDL, LDL, VLDL cholesterol; and triglycerides) and quantitative immunoturbidimetry for APOA-1, APOB, and Lp(a). Specimens were thawed, aliquoted, poured off into ARUP standard transport tube, refrozen, and delivered over dry ice to ARUP within 24 h of processing. All markers were directly measured by ARUP with the exception of LDL and VLDL, which were calculated using the Friedewald equation (LDL=Total Cholesterol - (HDL + VLDL)). VLDL was calculated as triglycerides/5. Prior studies have shown that storage at −70 °C or lower ensures long-term stability (≥10 years) for lipid measurements [12]. We additionally performed a validation test, comparing total cholesterol and triglyceride measurements measured within 3 months of collection (2007–2009) to measurements from banked samples (2024) from the same women (*n* = 401 women). We found high validity with a Pearson’s correlation of 0.99 (*p* < 0.001) for both cholesterol and triglycerides and kappa agreements of 0.94 (95 % CI: 0.91, 0.98) and 0.93 (95 % CI: 0.89, 0.98) for ≥200 vs <200 mg/dL cholesterol and ≥175 vs <175 mg/dL triglycerides. Standard at-risk cutpoints [13,14] for adult women were used for analysis including the following: total cholesterol: ≥200 mg/dL; HDL: <50 mg/dL; LDL: ≥100 mg/dL; triglycerides: ≥175 mg/dL; non-HDL: ≥130 mg/dL; VLDL: ≥30 mg/dL; ApoA-1: <125 mg/dL; APOB: ≥120 mg/dL; Lp(a): ≥30 mg/dL; and APOB B/A1: Ratio >0.78.

#### 2.3.4. Covariates

Factors known to influence women’s endometriosis and cardiometabolic health were considered potential confounders. We fit parsimonious regression models, adjusting for age at baseline, BMI, income (below poverty level vs above based upon the 2007 HHS Poverty Guidelines accounting for the numbers of persons in the household for the 48 contiguous states and District of Columbia.), and smoking (via serum cotinine). Additional confounders evaluated included caffeine and alcohol intake, as well as physical activity levels, and any prior report of hormonal contraceptive use. While parity and chronic/cyclic pelvic pain are most likely consequences of endometriosis, we hypothesized that they could also impact endometriosis development and thus we additionally ran models adjusting for these two factors.

### 2.4. Statistical analysis

Descriptive characteristics comparing women with and without endometriosis were generated, including n (%), means (SD), interquartile range (IQR) with median, and reporting missing values for covariates. Generalized linear models using Poisson regression with robust standard errors were used to calculate adjusted prevalence ratios (aPR) and 95 % confidence intervals (CIs) for the associations between endometriosis diagnosis, endometriosis staging (minimal/mild vs moderate/severe), and endometriosis typology (SE, OE, DE, and OE+DE) and prevalence of adverse lipid profiles. Given that lipid levels have been shown to significantly alter over the menstrual cycle, we also tested for effect modification by menstrual cycle phase (follicular versus luteal) in the relationship between endometriosis diagnosis, stage, and typology and adverse lipid profiles. We used the Wald test with a significance level of *P* < 0.20. Above analyses were conducted for both the operative and population cohorts for endometriosis diagnosis (yes/no). Analyses comparing endometriosis staging or endometriosis typology and adverse lipid profiles were only conducted in the operative cohort since surgically visualized disease is needed for endometriosis staging and typology. Given that missingness for main adjusted models was <5 %, complete case analyses were conducted.

## 3. Results

At the time of ENDO Study enrollment, participants were on average 32 years (SD: 7 years) with a BMI of 28 kg/m^2^ (SD=8). The majority were non-Hispanic white (79 %), married (71 %), and reported ever having using hormonal contraception (86 %). Women with, compared to without, endometriosis tended to be of higher income, married, and of normal weight (Table 1).

**Fig. 1.**
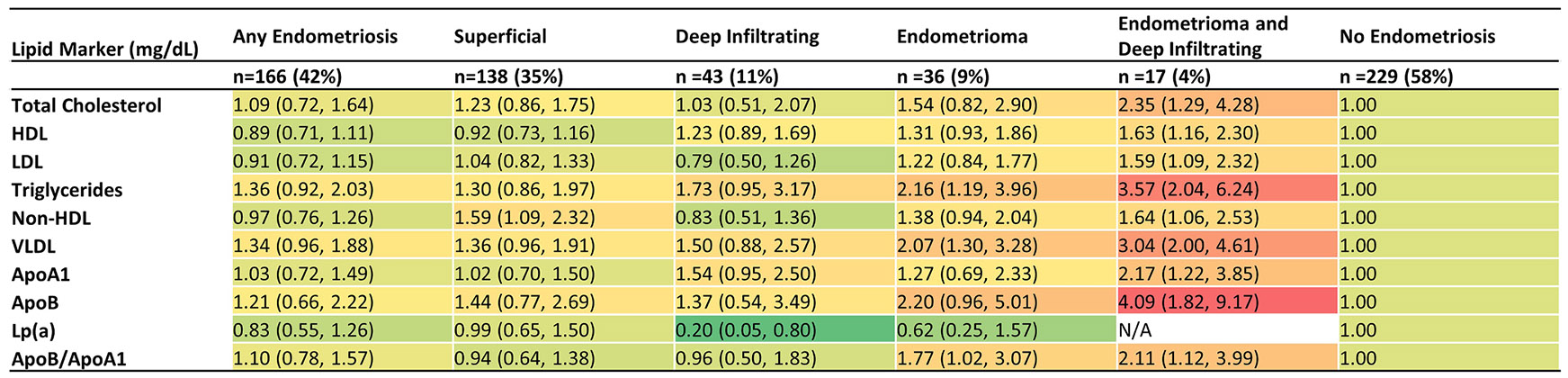
Heat map for adjusted prevalence ratios (95 % CI) of lipid marker cut points by endometriosis typology in ENDO operative cohort (*n* = 395). Adjusted for age at ENDO enrollment, BMI, serum cotinine, marital status, income, and race/ethnicity. Total cholesterol (≥200 mg/dL vs <200mg/dL); HDL: high-density lipoprotein (<50mg/dL vs ≥50mg/dL); LDL low-density lipoprotein (≥100mg/dL vs <100mg/dL); non-HDL: non-high-density lipoprotein (≥130 mg/dL vs <130 mg/dL); VLDL: very low-density lipoprotein (≥30 mg/dL vs <30mg/dL); ApoA1: Apolipoprotein A1 (<125 mg/dL vs ≥ 125mg/dL); ApoB: Apolipoprotein B (≥120 mg/dL vs <120 mg/dL; Lp(a): Lipoprotein(a) (≥30 mg/dL vs <30mg/dL); ApoB/ApoA1 ratio (>0.78 vs ≤ 0.78). Note, women with any endometriosis could have more than one type, thus the categories are not mutually exclusive.

Total mean cholesterol for women with endometriosis in the operative cohort was 178 ± 36 mg/dL (median: 176, IQR: 152, 197) with 23 % having total cholesterol ≥200 mg/dL (Table 2). Total mean cholesterol was similar for women without endometriosis in the operative cohort: mean=179 ± 33 mg/dL; median: 177, IQR: 157, 200) with 26 % having total cholesterol ≥200 mg/dL. Women with, versus without, endometriosis showed no association with levels of total cholesterol (aPR: 0.94, 95 % CI: 0.62, 1.37) in our fully adjusted model (Fig. 1). No appreciable patterns emerged for the other lipid markers for women with endometriosis compared to without endometriosis (Table 2, Fig. 1). Similar patterns were observed in the population cohort (Table 2).

A different pattern emerged when investigating endometriosis typology. Women with OE+DE, compared to no endometriosis, had higher prevalence of adverse lipid profiles: total cholesterol >200 mg/dL: 46 % vs 22 %; HDL cholesterol ⟨50mg/dL: 69 % vs 47 %; LDL cholesterol ≥100 mg/dL: 69 % vs 45 %; triglycerides ⟩ 175 mg/dL: 55 % vs 17 %; non-HDL cholesterol ≥130 mg/dL: 62 % vs 41 %; VLDL ≥30 mg/dL: 62 % vs 25 %; ApoA-1 <125 mg/dL: 47 % vs 24 %; APO-B mg/dL ≥120: 29 % vs 10 %; and APOB/ApoA-1 ratio: 40 % vs 24 % (Table 3). Using the same cut points, these differences held in multivariable models after adjusting for age at baseline, race/ethnicity, marital status, BMI, income (poverty level), and serum cotinine (Fig. 1). Women with OE+DE, compared to no endometriosis, had higher aPR of total cholesterol 2.35 (95 % CI: 1.29, 4.28); HDL 1.63 (95 % CI: 1.16, 2.30); LDL 1.59 (95 % CI: 1.09, 2.32); triglycerides 3.57 (2.04, 6.24); non-HDL 1.64 (95 % CI: 1.06, 2.53), VLDL 3.04 (2.00, 4.61); ApoA1 2.17 (95 % CI: 1.22, 3.85), ApoB 4.09 (95 % CI: 1.82, 9.17); and ApoB/ApoA1 ratio 2.11 (95 % CI: 1.12, 3.99). The only lipid marker to show lower prevalence among women with OE+DE compared to no endometriosis was Lp(a): 0 % vs 24 %. OE as compared to DE appeared to be driving the association between OE+DE and dyslipidemia (Fig. 1). SE was not associated with dyslipidemia (Fig. 1).

**Fig. 2.**
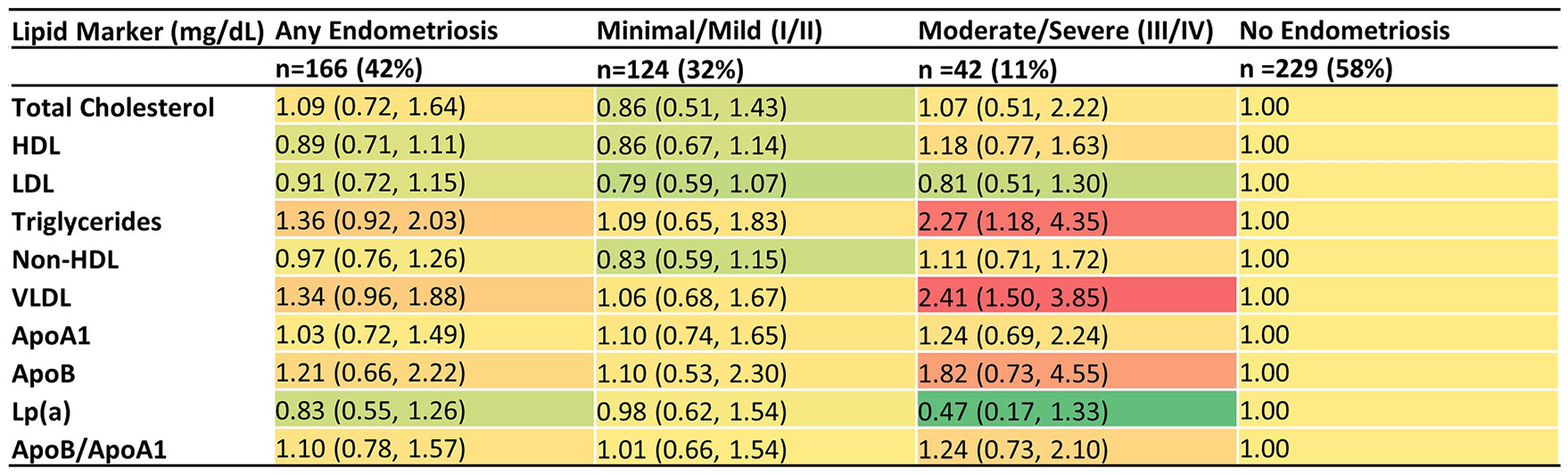
Heat map for adjusted prevalence ratios (95 % CI) of lipid marker cut points by endometriosis staging (Stage I/II=minimal/mild; Stage III/IV=moderate/severe) in ENDO operative cohort (*n* = 395). Adjusted for age at ENDO enrollment, BMI, serum cotinine, marital status, income, and race/ethnicity. Total cholesterol (≥200 mg/dL vs <200mg/dL); HDL: high-density lipoprotein (<50mg/dL vs ≥50mg/dL); LDL low-density lipoprotein (≥100mg/dL vs <100mg/dL); non-HDL: non-high-density lipoprotein (≥130 mg/dL vs <130 mg/dL); VLDL: very low-density lipoprotein (≥30 mg/dL vs <30mg/dL); ApoA1: Apolipoprotein A1 (<125 mg/dL vs ≥ 125mg/dL); ApoB: Apolipoprotein B (≥120 mg/dL vs <120 mg/dL)Lp(a): Lipoprotein(a) (≥30 mg/dL vs <30mg/dL); ApoB/ApoA1 ratio (>0.78 vs ≤ 0.78).

Regarding staging, a pattern emerged with women with moderate to severe endometriosis, compared to no endometriosis, having predominantly aPRs >1.0 for lipid markers (Table 4). Triglycerides and VLDL cholesterol showed the strongest associations, with aPRs of 2.27 (95 % CI: 1.18,4.35) and 2.41 (95 % CI: 1.50, 3.85) (Fig. 2).

Results for the association between endometriosis diagnosis, staging, and typology and lipid markers were similar when restricting the reference group to women without endometriosis and no other gynecologic pathology. Results did not appreciably alter when additionally adjusting for other potential confounders including caffeine and alcohol intake, physical activity levels, parity, and chronic/cyclic pelvic pain. We found no evidence for effect modification by menstrual cycle phase (all Wald>0.20) in the relationship between endometriosis diagnosis, staging, and typology and adverse lipid profiles.

## 4. Discussion

### 4.1. Principal findings

We observed that overall, endometriosis diagnosis was not associated with levels of lipid markers. However, we did observe a consistent pattern between endometriosis typology and lipid markers where women with endometriosis within their ovaries (endometriomas) and deep infiltrating endometriosis had a higher prevalence of elevated total cholesterol, LDL, triglycerides, non-HDL, and VLDL, and low HDL, which appeared to be driven primarily by endometriomas. We also observed that women with endometriomas and deep infiltrating endometriosis had higher prevalence of elevated ApoA1, ApoB, and ApoB/ApoA1 ratio.

### 4.2. Results in the context of what is known

The association between endometriosis and adverse lipid profiles has been investigated in a number of prior studies. Research from the Nurses’ Health Study II observed that a clinical diagnosis of endometriosis was associated with 25 % increased risk of a clinical diagnosis of hypercholesterolemia compared to those without endometriosis [4]. This association with elevated cholesterol has subsequently been confirmed in administrative health records [15], as have associations with hyperlipidemia [16]. When circulating cholesterol markers in relation to endometriosis have been investigated cross-sectionally, the literature is more mixed [6]. Some studies have observed higher total cholesterol, LDL, and HDL [7] for women with endometriosis compared to women without endometriosis, but this has not been consistent across all studies [6,8,17]. Possible differences in findings could be driven by small sample sizes (<50 participants with endometriosis) [6,7] or differences in patient age, temporality of lipid collection in relation to endometriosis diagnosis and treatment, and/or endometriosis categorization. We observed that women with OE and DE had 2.4 times the prevalence of elevated total cholesterol, 1.6 times the prevalence of high LDL, and 4.1 times the prevalence of high APO-B, which appeared to be driven primarily with associations with ovarian endometriomas (Total cholesterol aPR:1.5, LDL aPR:1.2, APO-B aPR: 2.2). Participants with endometriomas and deep infiltrating endometriosis were also observed to have an increased prevalence of lower HDL (PR:1.6).

Prior studies have observed that women with endometriosis have higher levels of triglycerides [5,7,18] compared to their counterparts. Mendelian randomization studies have observed associations with genetic scores of triglycerides and risk of endometriosis development [19]. When thinking about the possibility of causality in the opposite direction, some [20] but not all Mendelian randomization studies [21] have observed the association between endometriosis and risk of elevated triglycerides. Verit et al. observed that overall endometriosis diagnosis was associated with higher serum trigylcerides, which appeared to be stronger among women with moderate to severe endometriosis [5].

While we did not observe an association with endometriosis diagnosis overall and triglycerides, we did observe that participants with OE+DE had nearly a 3.6-fold increased prevalence of elevated triglycerides, which was higher for endometrioma (PR: 2.2) than deep infiltrating endometriosis (PR:1.7) when investigated separately.

In addition to directly measuring lipid levels among women with endometriosis, there has also been increasing research on endometriosis and lipid metabolites. Prior studies on circulating lipids levels among women diagnosed with endometriosis suggest aberrant lipid metabolomic profiles for women with endometriosis compared to their counterparts [18,22-25]. However, much of this research is among couples experiencing infertility with relatively small sample sizes, limiting their generalizability. Prior research has also observed that higher lipid metabolites were associated with risk of persistent pelvic pain one year following endometriosis surgery [26], suggesting that, consistent with our findings, there may be an association with endometriosis severity and aberrant lipids.

### 4.3. Clinical and research implications

Our findings indicate that lipid dysregulation may already be present at the time of endometriosis diagnosis, particularly among women with OE and DE. These subtypes may represent a pro-atherogenic endophenotype of endometriosis, potentially driven by shared pathways involving systemic inflammation, oxidative stress, and immune dysregulation [4,10]. Although routine lipid screening is not currently standard in endometriosis management, our findings suggest that lipid profiles may serve as markers of phenotype severity. Before translation to clinical guidelines, replication in larger and more diverse populations is needed, along with mechanistic studies to confirm causality, clarify the underlying biological processes, and ascertain whether lipid-lowering interventions or anti-inflammatory therapies may mitigate long-term CVD risk.

An additional dimension that may help explain these associations is familial and genetic susceptibility, which could represent a common upstream determinant linking endometriosis and cardiometabolic risk. Endometriosis is a heritable, chronic inflammatory disease with an estimated heritability of 47–52 % in twin studies [27,28]. Family history is a strong risk factor (sisters: relative risk ≈ 5.2; 95 % CI, 3.4–7.2), reflecting both genetic predisposition and shared environmental influences [29]. Genome-wide association studies have identified at least 12 single nucleotide polymorphisms across 10 loci, with the strongest associations observed in advanced rASRM stage III/IV disease (OE/DIE), suggesting potential subtype-specific genetic architecture [28,30]. These findings raise the possibility that the phenotype-specific lipid signal observed here—particularly elevated ApoB and triglycerides in OE/DIE—may partly reflect a shared inherited endophenotype influencing both endometriosis susceptibility and cardiometabolic programming. Future studies integrating genomic, epigenetic, and phenomic data will be critical to disentangle these relationships.

### 4.4. Strengths and limitations

Our study had several strengths including a study population with few exclusion criteria. This enhances generalizability, captures incident visualized endometriosis reducing misclassification bias, and assesses multiple confounding factors prior to surgery/MRI. This, in turn, limits residual confounding and recall bias of self-reported covariates. However, our study is not without limitations. Women who were part of the operative cohort were undergoing laparoscopy/laparotomy for multiple conditions. Therefore, the comparison group was not a random sample of “healthy women” and may have other conditions which may influence their lipid profiles. However, when restricting our operative comparison group to women without endometriosis and without other gynecologic pathology, our findings did not appreciably change. Additionally, the ENDO study was designed to have minimal exclusion criteria to maximize representativeness. Including women with other gynecologic indications reflects real-world clinical settings and enhances generalizability. However, external validation of findings outside of the Utah/U.S. population is needed to confirm generalizability. Regarding the association between endometriosis diagnosis and lipid markers, results were similar between the operative and population cohorts. An additional limitation was the non-fasting status of participants at the time of lipid marker sampling; however, prior research has shown that non-fasting triglyceride concentrations (the biomarker where we found some of our strongest effects) are a better predictor of cardiovascular risk than fasting triglycerides. Finally, our study had limited power in subgroup phenotypes, particularly for OE+DE, with only 17 women presenting with this severe phenotype. As a result, estimates for these subgroups may be unstable, and findings should be interpreted cautiously as hypothesis-generating rather than causal.

## 5. Conclusion

Overall, endometriosis diagnosis was not observed to be associated with levels of lipid markers. When we investigated endometriosis typology, however, we observed that endometriomas and deep infiltrating endometriosis were associated with increased prevalence of high total cholesterol, high triglycerides, high VLDL, and high APO-B. This association appears to be driven by endometriomas and a similar pattern was observed among higher stage (III/IV) endometriosis. Given that these biomarkers were collected concurrently with endometriosis diagnosis, this could indicate that a pattern of aberrant lipid markers is present in early adulthood, indicating that women with ovarian endometriomas and ovarian endometriomas plus deep infiltrating endometriosis may benefit from targeted screening or intervention in early to midlife.

## Figures and Tables

**Table 1:** Population characteristics by endometriosis status among ENDO Study participants in Utah inclusive of operative and population cohorts who had a baseline blood draw (*n* = 476 total comprising 395 in operative cohort and 81 in population cohort).

Population Characteristics	Overall *N* = 476	Endometriosis n (%)	
		Yes (*n* = 178)	No (*n* = 298)
Age at visit (yrs.), Mean ± SD	32.2 ± 7.1	31.6 ± 6.90	32.63 ± 7.27
Race/Ethnicity, n (%)			
Hispanic	58 (12.2)	23 (12.9)	35 (11.7)
Non-Hispanic White	377 (79.2)	139 (78.1)	238 (79.9)
Non-Hispanic Black	4 (0.8)	1 (0.6)	3 (1.0)
South Asian	20 (4.2)	8 (4.5)	12 (4.0)
Multi-racial	9 (1.9)	2 (1.1)	7 (2.4)
Other	8 (1.7)	5 (2.8)	3 (1.0)
Income, n (%)			
Below Poverty Line	63 (13.4)	19 (10.9)	44 (14.9)
≤180 % of Poverty Line	55 (11.7)	11 (6.3)	44 (14.9)
>180 % of Poverty Line	353 (75.0)	145 (82.9)	208 (70.3)
Marital Status, n (%)s			
Married	334 (70.6)	131 (74.0)	203 (68.6)
Single, Living as married	21 (4.4)	7 (4.0)	14 (4.7)
Other	118 (25.0)	39 (22.0)	79 (26.7)
BMI (Kg/m^2^), n (%)			
Underweight (<18.5)	22 (4.6)	11 (6.2)	11 (3.7)
Normal Weight (18.5–24.99)	184 (38.7)	83 (46.6)	101 (33.9)
Overweight (25–29.99)	111 (23.3)	42 (23.6)	69 (23.2)
Obese (≥30.0)	159 (33.4)	42 (23.6)	117 (39.3)
Ever taken hormonal birth control, n (%)	409 (86.0)	156 (87.6)	253 (84.9)
Serum Cotinine, Median (IQR)			
25th Percentile	0.004	0.003	0.006
75th Percentile	0.09	0.07	0.14
Menstrual Cycle Phase, n (%)			
Follicular	205 (51.5)	78 (51.0)	127 (51.8)
Luteal	193 (48.5)	75 (49.0)	118 (48.2)

Missing values: *n* = 1 age at visit, *n* = 0 for race; *n* = 5 for income, *n* = 3 for marital status, *n* = 5 BMI; *n* = 0 serum cotinine; *n* = 78 menstrual cycle phase.

**Table 2:** Lipid markers by endometriosis status in operative and population cohort (*n* = 476 total made up of *n* = 395 in operative cohort and 81 in population cohort).

Lipid Marker (mg/dL)	Operative Cohort		Population	
	Endometriosis *N* = 166 (42 %)	No Endometriosis *n* = 229 (58 %)	Endometriosis *n* = 12 (15 %)	No Endometriosis *n* = 69 (85 %)
Total Cholesterol, Mean ± SD	177.59 ± 35.93;	179.43 ± 32.99	181.89 ± 36.60	186.35 ± 34.81
Median (25 %,75 %)	176 (152, 197)	177 (157, 200)	167 (159, 207)	181 (165, 212)
≥200, n (%)	30 (23)	50 (26)	3 (33)	16 (33)
HDL, Mean ± SD	51.11 ± 12.95	49.34 ± 12.20	49.22 ± 11.73	50.18 ± 12.93
Median (25 %,75 %)	52 (42, 59)	48 (40, 56)	55 (42, 58)	49 (39, 57)
<50, n (%)	60 (45)	108 (55)	4 (44)	26 (53)
LDL, Mean ± SD	98.53 ± 31.28	104.50 ± 30.12	107.22 ± 39.70	106.26 ± 31.93
Median (25 %,75 %)	96 (74, 121)	102 (84, 124)	89 (81, 138)	106 (85, 132)
≥100, n (%)	58 (44)	104 (53)	4 (44)	28 (60)
Triglycerides, Mean ± SD	137.05 ± 77.88	130.94 ± 76.65	127.78 ± 48.10	158 ± 107.92
Median (25 %,75 %)	111 (83, 168)	112.5 (83, 157.5)	115 (88, 162)	139 (86, 166)
≥175, n (%)	31 (25)	38 (21)	2 (25)	12 (29)
Non-HDL, Mean ± SD	126.47 ± 38.11	130.09 ± 34.08	132.67 ± 47.32	136.16 ± 39.65
Median (25 %,75 %)	119 (98, 152)	127.5 (105.5, 149.5)	106 (99, 161)	135 (110, 162)
≥130, n (%)	54 (41)	93 (48)	4 (44)	26 53)
VLDL, Mean ± SD	26.45 ± 13.45	25.53 ± 12.21	25.44 ± 9.50	28.51 ± 15.53
Median (25 %,75 %)	22 (17, 34)	22 (17, 31)	23 (18, 32)	28 (17, 33)
≥30, n (%)	40 (31)	56 (29)	3 (33)	17 (36)
ApoA1, Mean ± SD	143.59 ± 27.67	139.97 ± 25.54	137.36 ± 15.32	143.52 ± 24.56
Median (25 %,75 %)	140 (124, 159)	136.5 (122, 155)	142 (127, 146)	139 (128, 160)
<125, n (%)	39 (25)	55 (26)	2 (18)	15 (23)
ApoB, Mean ± SD	88.9 ± 23.20	90.06 ± 23.50	79 ± 23.60	92.46 ± 25.86
Median (25 %,75 %)	86 (74, 100)	88 (74, 105)	70.5 (62.5, 96)	91 (74, 112)
≥120, n (%)	16 (11)	22 (11)	1 (13)	8 (13)
Lp(a), ≥30, n (%)	30 (22)	54 (26)	5 (40)	10 (20)
ApoB/ApoA1 >0.78, n (%)	36 (25)	56 (29)	2 (29)	21 (36)

**Table 3:** Lipid markers by endometriosis typology in ENDO Study participants in Utah operative cohort (*n* = 395).

Lipid Marker (mg/dL)	Superficial *n* = 138 (35 %)	Deep Infiltrating *n* =43 (11 %)	Endometrioma *n* = 36 (9 %)	Endometrioma and Deep Infiltrating *n* = 17 (4 %)	No Endometriosis *n* = 229 (58 %)
Total Cholesterol, Mean ± SD	180 ± 36	173 ± 32	181 ± 33	194 ± 25	179 ± 33
Median (25 %,75 %)	180 (155, 198)	175 (155, 196)	183 (156, 210)	196 (175, 219)	177 (157, 200)
≥200, n (%)	27 (24)	7 (21)	8 (30)	6 (46)	50 (22)
HDL, Mean ± SD	50 ± 12	50 ± 13	49 ± 14	46 ± 12	49 ± 12
Median (25 %,75 %)	51 (41, 57)	49 (39, 60)	47 (38, 56)	45 (36, 56)	48 (40, 56)
<50, n (%)	52 (46)	18 (55)	15 (56)	9 (69)	108 (47)
LDL, Mean ± SD	102 ± 30	93 ± 27	102 ± 33	112 ± 26,	105 ± 30
Median (25 %,75 %)	98 (81, 123)	94 (74, 109)	103 (74, 128)	106 (99, 130)	102 (84, 124)
≥100, n (%)	53 (48)	12 (36)	14 (51)	9 (69)	104 (45)
Triglycerides, Mean ± SD	138 ± 80	147 ± 81	151 ± 83	179 ± 87	131 ± 76
Median (25 %,75 %)	110 (83, 168)	121 (88, 185)	125 (83, 218)	172 (117, 218)	113 (83, 158)
≥175, n (%)	25 (25)	9 (29)	9 (38)	6 (55)	38 (17)
Non-HDL, Mean ± SD	130 ± 37	123 ± 34	132 ± 40	148 ± 31	130 ± 34
Median (25 %,75 %)	122 (104, 154)	119 (97, 137)	131 (110, 165)	150 (119, 172)	128 (106, 150)
≥130, n (%)	47 (42)	11 (33)	14 (52)	8 (62)	93 (41)
VLDL, Mean ± SD	26 ± 14	29 ± 16	30 ± 17	36 ± 17	26 ± 12
Median (25 %,75 %)	22 (17, 33)	24 (18, 37)	25 (17, 44)	34 (23, 44)	22 (17, 31)
≥30, n (%)	34 (31)	11 (33)	12 (44)	8 (62)	56 (25)
ApoA1, Mean ± SD	142 ± 26	143 ± 28	142 ± 28	141 ± 32	140 ± 26
Median (25 %,75 %)	140 (124, 158)	143 (121, 152)	136 (124, 153)	133 (114, 152)	137 (122, 155)
<125, n (%)	33 (26)	14 (37)	9 (27)	7 (47)	55 (24)
ApoB, Mean ± SD	90 ± 24	88 ± 22	97 ± 23	104 ± 23	90 ± 24
Median (25 %,75 %)	86 (75, 101)	89 (71, 96)	96 (84, 109)	96 (89, 122)	88 (74, 105)
≥120, n (%)	15 (12)	5 (12)	6 (18)	5 (29)	22 (10)
Lp(a), ≥30, n (%)	28 (24)	3 (8)	4 (15)	0 (0)	54 (24 %)
ApoB/ApoA1 >0.78, n (%)	32 (26)	8 (21)	11 (35)	6 (40)	56 (24)

**Table 4:** Lipid markers by endometriosis staging in ENDO Study participants in Utah operative cohort (*n* = 395).

Lipid Marker (mg/dL)	Minimal/Mild (Stage I/II) *n* = 124 (32 %)	Moderate/ Severe (Stage III/IV) *n* = 42 (11 %)	No Endometriosis *n* = 229 (58 %)
Total Cholesterol, Mean ± SD	176 ± 37	182 ± 32	179 ± 33
Median (25 %,75 %)	175 (150, 195)	185 (157, 209)	177 (157, 200)
≥200, n (%)	21 (21)	9 (28)	50 (22)
HDL, Mean ± SD	52 ± 13	49 ± 13	49 ± 12
Median (25 %,75 %)	53 (42, 59)	48 (39, 59)	48 (40, 56)
<50, n (%)	41 (41)	19 (59)	108 (47)
LDL, Mean ± SD	98 ± 32	100 ± 29	105 ± 30
Median (25 %,75 %)	95 (74, 118)	99 (78, 125)	102 (84, 124)
≥100, n (%)	43 (43)	15 (47)	104 (45)
Triglycerides, Mean ± SD	127 ± 72	167 ± 87	131 ± 76
Median (25 %,75 %)	109 (82, 146)	150 (89, 240)	113 (83, 158)
≥175, n (%)	18 (18)	13 (41)	38 (17)
Non-HDL, Mean ± SD	124 ± 38	133 ± 37	130 ± 34
Median (25 %,75 %)	116 (97, 148)	133 (112, 161)	128 (106, 150)
≥130, n (%)	37 (37)	17 (53)	93 (41)
VLDL, Mean ± SD	24 ± 11	34 ± 17	26 ± 12
Median (25 %,75 %)	21 (16, 29)	30 (18, 48)	22 (17, 31)
≥30, n (%)	23 (23)	17 (53)	56 (25)
ApoA1, Mean ± SD	144 ± 27	143 ± 29	140 ± 26
Median (25 %,75 %)	141 (126, 161)	138 (124, 154)	137 (122, 155)
<125, n (%)	28 (25)	11 (28)	55 (24)
ApoB, Mean ± SD	87 ± 23	95 ± 22	90 ± 24
Median (25 %,75 %)	83 (71, 99)	92 (82, 109)	88 (74, 105)
≥120, n (%)	10 (9)	6 (15)	22 (10)
Lp(a), ≥30, n (%)	26 (25)	4 (13)	54 (24 %)
ApoB/ApoA1 >0.78, n (%)	41 (33)	15 (36)	56 (24)

## Data Availability

Data will be made available to the editors of the journal for review or query on request and after appropriate approvals from data steward *Eunice Kennedy Shriver National Institute of Child Health and Human Development* have been secured.
